# Characterization of *Aquifex aeolicus* 4-diphosphocytidyl-2*C*-methyl-d-erythritol kinase – ligand recognition in a template for antimicrobial drug discovery

**DOI:** 10.1111/j.1742-4658.2008.06418.x

**Published:** 2008-06

**Authors:** Tanja Sgraja, Magnus S Alphey, Stephanos Ghilagaber, Rudi Marquez, Murray N Robertson, Jennifer L Hemmings, Susan Lauw, Felix Rohdich, Adelbert Bacher, Wolfgang Eisenreich, Victoria Illarionova, William N Hunter

**Affiliations:** 1 Division of Biological Chemistry and Drug Discovery, University of DundeeUK; 2 Department of Chemistry, University of GlasgowUK; 3 Center for Integrated Protein Research, Technische Universität MünchenGarching, Germany

**Keywords:** enzyme–ligand complex, GHMP kinase, isoprenoid biosynthesis, molecular recognition, non-mevalonate pathway

## Abstract

4-Diphosphocytidyl-2*C*-methyl-d-erythritol kinase (IspE) catalyses the ATP-dependent conversion of 4-diphosphocytidyl-2*C*-methyl-d-erythritol (CDPME) to 4-diphosphocytidyl-2*C*-methyl-d-erythritol 2-phosphate with the release of ADP. This reaction occurs in the non-mevalonate pathway of isoprenoid precursor biosynthesis and because it is essential in important microbial pathogens and absent from mammals it represents a potential target for anti-infective drugs. We set out to characterize the biochemical properties, determinants of molecular recognition and reactivity of IspE and report the cloning and purification of recombinant *Aquifex aeolicus* IspE (*Aa*IspE), kinetic data, metal ion, temperature and pH dependence, crystallization and structure determination of the enzyme in complex with CDP, CDPME and ADP. In addition, 4-fluoro-3,5-dihydroxy-4-methylpent-1-enylphosphonic acid (compound **1**) was designed to mimic a fragment of the substrate, a synthetic route to **1** was elucidated and the complex structure determined. Surprisingly, this ligand occupies the binding site for the ATP α-phosphate not the binding site for the methyl-d-erythritol moiety of CDPME. Gel filtration and analytical ultracentrifugation indicate that *Aa*IspE is a monomer in solution. The enzyme displays the characteristic α/β galacto-homoserine-mevalonate-phosphomevalonate kinase fold, with the catalytic centre positioned in a deep cleft between the ATP- and CDPME-binding domains. Comparisons indicate a high degree of sequence conservation on the IspE active site across bacterial species, similarities in structure, specificity of substrate recognition and mechanism. The biochemical characterization, attainment of well-ordered and reproducible crystals and the models resulting from the analyses provide reagents and templates to support the structure-based design of broad-spectrum antimicrobial agents.

Isoprenoids are one of the largest groups of natural products and include primary and secondary metabolites such as sterols, dolichols and triterpenes, the prenyl groups of chlorophyll and modified proteins. Isoprenoids contribute to important biological functions including respiration, photosynthesis, hormone-based signalling, apoptosis, meiosis and protein degradation, and in addition, provide important structural components of cell membranes [1–3].

Two biosynthetic pathways to isopentenyl diphosphate and dimethylallyl disphosphate, the basic five-carbon precursors for all isoprenoids, have evolved. The mevalonate pathway, named after one of its constituent metabolites, is found in archaebacteria, some eubacteria, yeast, the cytosolic compartment of plants and animals. In chloroplasts, algae, cyanobacteria, most eubacteria and the apicomplexa, the biosynthesis of isopentenyl diphosphate and dimethylallyl disphosphate is via the non-mevalonate pathway [4–7]. Species that utilize and are dependent on the non-mevalonate pathway include the causal agents for important human diseases such as leprosy, tuberculosis and malaria. The enzymes of this pathway are absent from humans and have attracted interest as potential targets for antimicrobial drug screening [8], structure-based drug discovery and design [4].

Our interest centres on 4-diphosphocytidyl-2*C*-methyl-d-erythritol kinase (IspE, EC 2.7.1.148), which catalyses the transfer of the ATP γ-phosphate to 4-diphosphocytidyl-2*C*-methyl-d-erythritol (CDPME) forming 4-diphosphocytidyl-2*C*-methyl-d-erythritol 2-phosphate (CDPME2P) and ADP (Fig. 1) [9,10]. IspE is a GHMP kinase, named after the four founding members of this protein superfamily namely galacto, homoserine, mevalonate and phosphomevalonate kinases [11,12]. The crystal structure of the *Escherichia coli* orthologue (*Ec*IspE) in complex with a stable ATP analogue, adenosine 5′-(β,γ-imino)triphosphate (AMP-PNP) and CDPME, as well as the apo-structure of the *Thermus thermophilus* enzyme (*Tt*IspE) have been reported [13,14].

**Fig. 1:**
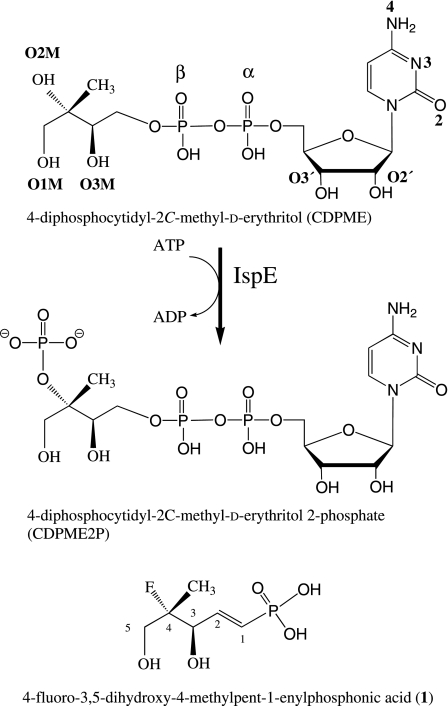
The reaction catalysed by IspE together with the structure of 1 to highlight similarity with part of the substrate.

Research with IspE from several organisms has been hampered because the recombinant proteins have low solubility. Here, we show that IspE from the thermophilic bacterium *Aquifex aeolicus* (*Aa*IspE) provides an efficient expression system of a stable and soluble recombinant enzyme. This has allowed, for the first time, detailed biochemical and kinetic characterization of IspE, determination of the metal ion and temperature dependence and optimum pH. The enzyme gives well-ordered crystals and we applied multiple-wavelength anomalous dispersion phasing methods to a selenomethionine (SeMet) derivative to solve the structure. Complexes with cytidine, CDP, CDPME, ADP and the newly synthesized ligand, 4-fluoro-3,5-dihydroxy-4-methylpent-1-enylphosphonic acid (**1**), are reported.

## Results and Discussion

### Biochemical and kinetic characterization

The putative *ispE* gene from *A. aeolicus* was placed under the control of the T_7_ promoter and lac operator in the hyperexpression plasmid pET-15b, and heat transformed into *E. coli* BL21 (DE3). The gene was preceded by a His_6_ tag to enable purification of the recombinant protein via metal-chelating affinity chromatography. The polyhistidine tag was removed by thrombin-mediated proteolysis, followed by purification with anion-exchange chromatography. The protein was purified to homogeneity with a yield of ∼ 15 mg protein per litre of cell culture. The protein had an apparent mass of 30 kDa, as judged by gel-filtration chromatography, which is in a good agreement with a sedimentation velocity experiment affording a calculated mass of 29.8 kDa (data not shown).

**Fig. 2:**
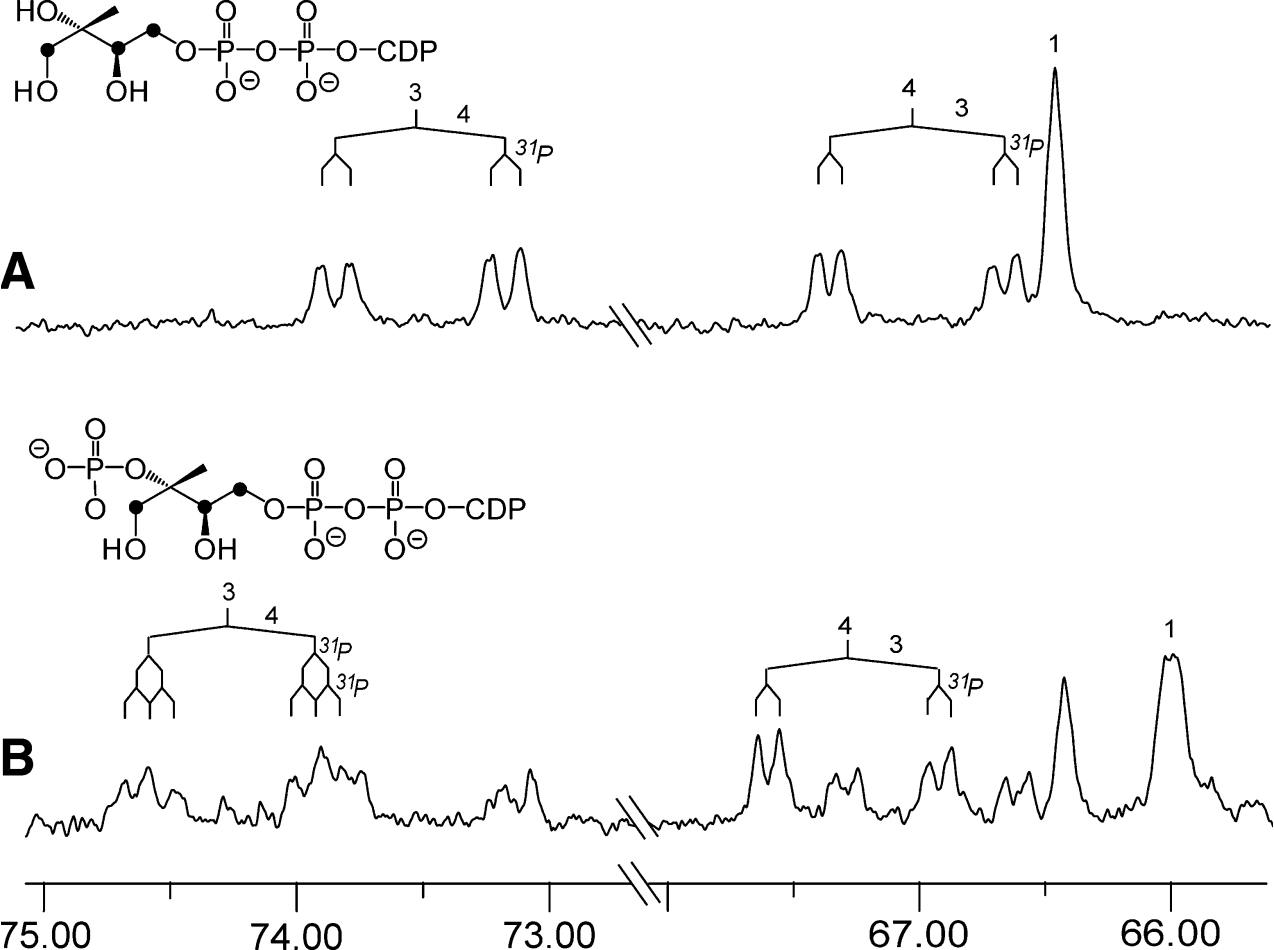
^13^C NMR spectra of an enzymatic assay with *Aa*IspE. (A) Signals of [1,3,4-^13^C_3_]CDPME before incubation; (B) 65% conversion from [1,3,4-^13^C_3_] CDPME into [1,3,4-^13^C_3_] CDPME2P. The ^13^C NMR signals of the substrate and the product agree published data [8].

**Fig. 3:**
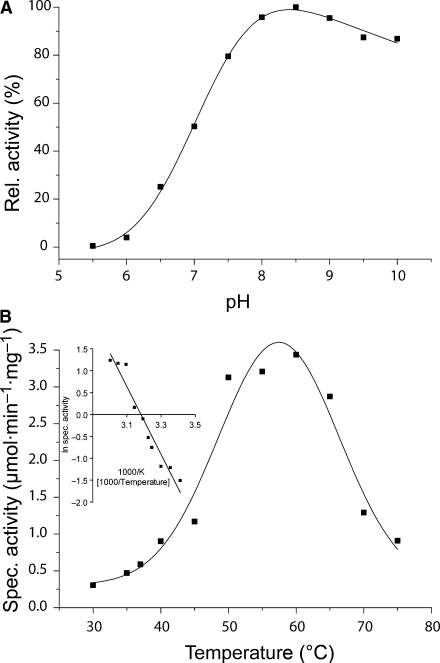
pH and temperature dependence of *Aa*IspE. (A) Relative activity versus pH. The measurements were performed using the photometric assay as described in Experimental procedures in a buffer containing 50 mm citrate, 50 mm Hepes, 50 mm Tris/HCl and 50 mm boric acid in a total volume of 200 μL. pH was adjusted to values of 5-10 with hydrochloric acid and sodium hydroxide, respectively. (B) Specific activity at different temperatures, inset: Arrhenius plot. The measurements were performed using the NMR assay as described in Experimental procedures.

**Fig. 4:**
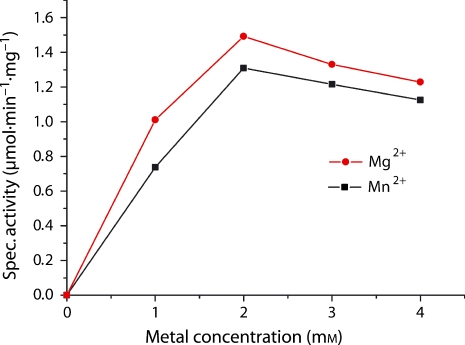
Mg^2+^ and Mn^2+^ dependence. The optimum concentration of both metals is 2 mm.

**Fig. 5:**
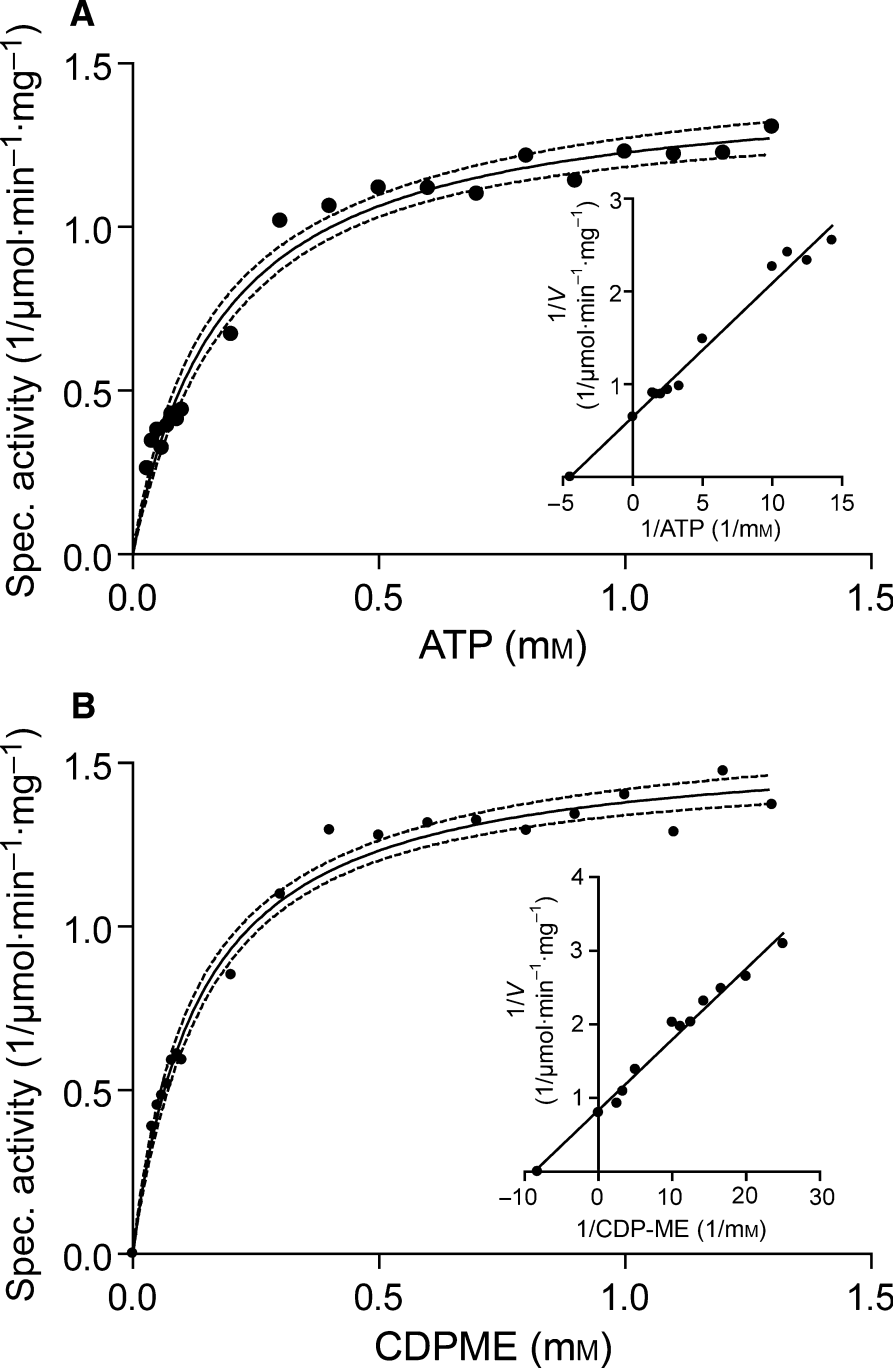
Substrate dependence of *Aa*IspE. (A) Michaelis–Menten kinetics showing initial rate versus ATP concentration. (B) Michaelis–Menten kinetics showing initial rate versus CDPME concentration; insets, Lineweaver–Burk; dotted lines show 95% confidence. Measurements were performed using the photometric assay as described in Experimental procedures. The concentration of ATP and CDPME were varied from 10 to 1300 μm, respectively.

The catalytic activity of the recombinant IspE protein was measured by ^13^C NMR spectroscopy and photometry. The ^13^C NMR measurement was performed using the multiply ^13^C-labelled substrate [1,3,4-^13^C_3_]CDPME to enhance the sensitivity and selectivity of ^13^C observation. ^13^C NMR signals detected in a typical NMR assay are shown in Fig. 2. The chemical shifts as well as the ^13^C^13^C-coupling constants were in excellent agreement with the published data on CDPME [15] and CDPME2P [9]. Optimal activity was identified at pH 8.5 and ∼ 60 °C (Fig. 3). *Aa*IspE was catalytically active in the presence of various metal ions (Table 1), and a highest rate of 1.5 μmol·min^−1^·mg^−1^ was achieved in the presence of 2 mm Mg^2+^ or Mn^2+^ (Fig. 4). The photometric assay was performed according to a published procedure [8] using auxiliary enzymes to observe the decrease in NADH absorption at 340 nm. The *K*_M_ values for CDPME and ATP were determined as 121 and 222 μm, respectively (Table 2, Fig. 5). The data are similar to the kinetic properties reported for IspE from *E. coli* [16] and tomato [17], respectively.

**Table 1:** Metal ion dependence of *Aa*IspE. Measurements were performed using the NMR assay as described in Experimental procedures with 5 mm divalent metal ions.

Metal	Relative activity (%)
None	0
Mg^2+^	100
Mn^2+^	94
Co^2+^	52
Cu^2+^	40
Fe^2+^	16
Zn^2+^	14
Ni^2+^	11
Ca^2+^	8

**Table 2:** Kinetic parameters of *Aa*IspE.

Kinetic parameters	Values
V_max_ at 37 °C	1.5 μmol·min^−1^·mg^−1^
*K*_m_ of 4-diphosphocytidyl-2-C-methyl-d-erythritol	121 μm
*K*_m_ of ATP	222 μm
Metal of choice	Mn^2+^, Mg^2+^
pH optimum	8.5
Temperature maximum	60 °C
Activation energy	64.2 kJ·mol^−1^

### Overall structure of AaIspE

Ordered and reproducible crystals of *Aa*IspE were obtained and multiple-wavelength anomalous dispersion methods were applied to obtain the initial phases. Subsequently, three medium-resolution complex crystal structures were determined (Tables 3 and 4). The crystals are isomorphous with two molecules, total molecular mass ∼ 60 kDa, in the asymmetric unit. The molecules, A and B, are related by a non-crystallographic twofold axis (data not shown). The surface area between the two molecules is ∼ 540 Å per molecule, ∼ 4% of the total surface area of the protein. This low value is consistent with gel-filtration and ultracentrifugation experiments that indicate *Aa*IspE is a monomer in solution (data not shown).

**Table 3:** Multiple-wavelength anomalous dispersion data statistics. Numbers in parentheses are for the highest resolution shell, a bin of 0.15 Å.

	λ_1_	λ_2_	λ_3_
Observed/unique reflections	219 842/24 238	220 860/24 266	222 261/24 265
Completeness (%)	100.0 (100.0)	100.0 (100.0)	100.0 (100.0)
Multiplicity/*R*_merge_ (%)	9.1 (8.6)/10.8 (44.2)	9.1 (8.6)/10.5 (42.9)	9.2 (8.9)/10.5 (43.9)
<I/σ (I)>	16.9 (4.2)	17.9 (4.4)	17.6 (4.3)

There are two molecules per asymmetric unit and therefore the three crystal structures provide six crystallographically independent molecules. These molecules are highly similar. Pair-wise least-squares superpositions give a range of r.m.s.d. of 0.17–0.46 Å for all Cα positions with a mean of 0.33 Å. Only minor differences are observed in the conformation of flexible side chains on α6 and the loop region following α4. The binding mode of the ligands differs only slightly within the asymmetric units and will be described in detail when applicable.

**Table 4:** Crystallographic statistics. Numbers in parentheses represent the highest resolution bin of width ∼ 0.06 Å.

	Complex I	Complex II	Complex III
Space group	*P*2_1_3	*P*2_1_3	*P*2_1_3
Unit cell length *a = b = c* (Å)	137.1	136.9	137.2
Resolution range (Å)	40–2.1	40.0–2.25	40.0–2.30
No. observed/unique reflections	262 443/49 871	304 071/40 884	285 651/38 442
Wilson *B* (Å^2^)	32.8	37.8	39.4
Completeness (%)	99.4 (95.7)	100.0 (100.0)	100.0 (100.0)
Multiplicity/*R*_merge_ (%)	5.3 (4.1)/6.4 (45.2)	7.4 (7.6)/13.7 (49.1)	7.4 (7.4)/11.6 (50.4)
<I/σ(I)>	17.1 (3.1)	12.1 (2.9)	14.2 (3.3)
*R*_work_/*R*_free_ (%)	20.8 (28.3)/25.2 (33.8)	20.2 (24.8)/22.9 (29.6)	19.7 (24.3)/24.5 (32.4)
r.m.s.d. from ideal bond lengths (Å)/angles (°)	0.008/1.186	0.008/1.168	0.008/1.096
Average *B* values (Å^2^)			
Overall/main chain/ side chain/waters/ ligands	30.9/30.4/31.4/38.8 (2 CDP 30.7; 2 ADP 43.8; 4 SO_4_^2−^ 53.9; 2 Cl^−^ 23.1)	30.9/30.3/31.4/36.0 (2 CDPME 30.9; 2 ADP 39.7; 3 SO_4_^2−^ 59.4; 3 Cl^−^ 28.0)	30.0/29.5/30.5/37.9 (2 CTN 41.1; 1 ligand 43.2; 8 SO_4_^2−^ 54.9; 2 Cl^−^ 19.5)
Ramachandran plot analysis			
Most favourable regions (%)	94.3	94.9	94.0
Additional allowed regions (%)	5.3	4.6	5.5
DPI^a^ (Å)	0.20	0.23	0.25

^a^ Diffraction-Component Precision Index [43].

**Fig. 6:**
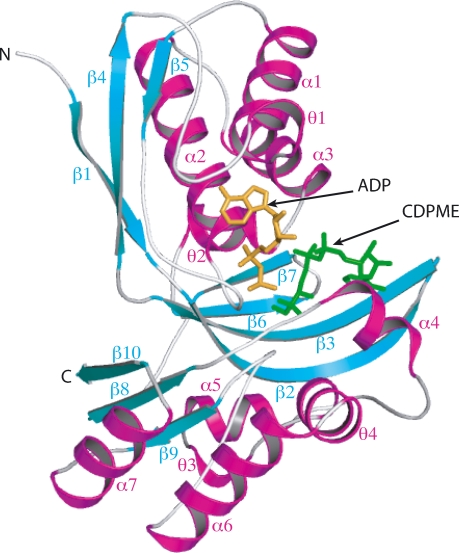
Ribbon diagram of *Aa*IspE. α and 3_10_ helices are red and β strands are cyan. Secondary structure elements have been labelled, N- and C-termini are marked. ADP is gold, the substrate CDPME green.

*Aa*IspE displays the typical GHMP kinase fold that comprises two domains (Fig. 6) [13]. The N-terminal domain (residues 1–155) consists of an elongated six-stranded β sheet (β1–β6). In addition, the concave side of the β sheet is flanked by three α helices (α1–α3) and two 3_10_ helices (θ1 and θ2). The C-terminal domain comprises an antiparallel four-stranded β sheet (β7–β10), bordering β1 and β2 of the N-terminal domain, four α helices (α4–α7) and two 3_10_ helices (θ3 and θ4).

**Fig. 7:**
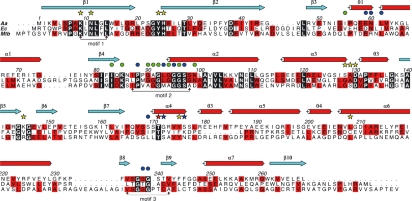
Primary and secondary structure of *Aa*IspE and sequence alignment of *A. aeolicus* (*Aa*), *E. coli* (*Ec*) and *M. tuberculosis* (*Mtb*) orthologues. Residues conserved in all three sequences are boxed in black, those conserved in two of the three sequences are boxed in red. Three GHMP kinase superfamily conserved motifs are marked. The secondary structure of *Aa*IspE is shown with helices as red cylinders and strands as cyan arrows. Residues marked with a star interact with substrate (yellow indicates a direct interaction, blue interactions bridged by water molecules). Thr171 is marked with a red star to indicate that it interacts with both CDPME and ADP. Residues marked with a dot interact with ADP (green indicates a direct interaction, blue water mediated interactions).

GHMP kinases have three conserved motifs that help create the catalytic centre [12,18,19]. In *Aa*IspE (Fig. 7) the residues of motif 1 (Lys9 to Leu14) are on β1 of the N-terminal domain and interact with CDPME, residues of motif 2 (Ile87 to Ser98) and motif 3 (Val235 to Val242), interact with the triphosphate component of ATP (see later). Motif 3 comprises the β8–β9 loop, which does not interact with the ligands but stabilizes the conformation of motifs 1 and 2 via hydrogen-bonding contacts. For example, Ser236 OG and Gly237 N form contacts with Asn11 OD1 of motif 1, Ser236 OG interacts with Gly92 O of motif 2, whereas Ser240 N and Thr241 OG1 donate hydrogen bonds to Gly90 O and Ala91 O respectively (not shown). Motif 2 is part of the β4–α2 loop, commonly described as the phosphate binding or P-loop. The main-chain amides of this glycine-rich motif and the α3 dipole surround the negatively charged phosphates of the nucleotide. The details of interactions formed between the enzyme motifs together with additional parts of the active site and the ligands are presented after comparison with structures of IspE orthologues.

### Comparison of IspE structures

**Fig. 8:**
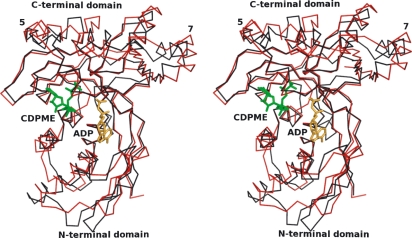
Stereo-view Cα overlay of *Aa*IspE and *Ec*IspE. *Aa*IspE is shown in red with *Ec*IspE in black. Also depicted are the ADP (gold) and CDPME (green) from the *Aa*IspE complex I. The positions of α5 and α7 are marked.

*Aa*IspE shares the highest sequence identity with *Tt*IspE (32%) and slightly less with *Ec*IspE (30%). The alignment of *Ec*IspE with *Aa*IspE in shown in Fig. 7. The DALI server [20] identified *Ec*IspE (*Z*-score: 29.1) and *Tt*IspE (*Z*-score: 27.7) as most similar to *Aa*IspE. (The *Z*-score is a measure of the statistical significance of the best subunit–subunit alignment and was determined by DALI. Typically, dissimilar proteins will have a *Z*-score < 2.0.) Cα superpositions of *Ec*IspE and *Tt*IspE with *Aa*IspE give r.m.s.d values of 1.6 and 1.8 Å, respectively, which mainly correspond to differences in the C-terminal domain distant from the active site. An overlay of *Aa*IspE and *Ec*IspE structures is depicted in Fig. 8. In general, the fold and secondary structure are well conserved. With respect to *Aa*IspE, α5 and α7 are situated at different positions in *Ec*IspE (Fig. 8) and *Tt*IspE structures, and an additional helix following β10 is inserted in *Ec*IspE. The two structures are further decorated with a 3_10_ helix following α7 or α4 respectively (not shown). The structural comparison and alignment of *Aa*IspE with *Ec*IspE reveals a high degree of conservation within the active site (Fig. 8). Alignment of the *Mycobacterium tuberculosis* IspE (*Mtb*IspE) sequence with those of *Aa*IspE and *Ec*IspE indicates sequence identities of around 25% (Fig. 7). This comparison is detailed later.

### The substrate-binding site

Three crystal structures of *Aa*IspE in complex with ligands were determined. Although data for complex I were measured from *Aa*IspE co-crystallized with the relatively stable ATP analogue AMP-PNP only ADP could be modeled. Similarly, CDPME2P was present in the crystallization conditions for the sample used to derive complex II, but only CDPME was observed. The lack of electron density for the terminal phosphate groups of CDPME2P and AMP-PNP is either a result of disorder and/or hydrolysis. In complex III, cytidine, ADP and **1** are present. The structures and interactions formed between *Aa*IspE and the ligands are similar in each complex and we concentrate on the details of complex II to indicate how substrate and ADP bind and then describe the specifics of interactions with **1**.

**Fig. 9:**
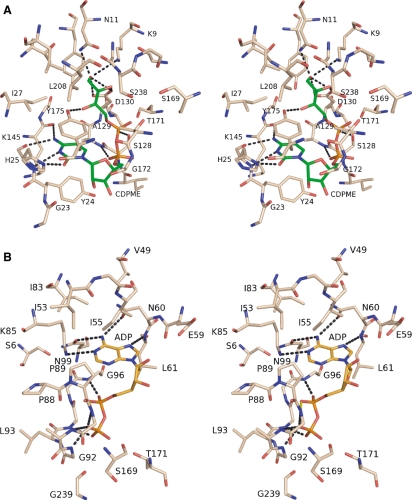
IspE ligand interactions in complex I. In both sections of this figure atoms are coloured blue N, red O and orange P. The C atoms of protein are beige, of CDPME green and ADP yellow. Residues that interact with the ligands are labelled, water molecules have been omitted for the purpose of clarity and some of the potential hydrogen bonds are shown as black dashed lines. (A) Stereo-view of the CDPME-binding site. (B) Stereo-view of the ADP-binding site.

The substrate CDPME binds into a deep cavity between the N- and C-terminal domains, surrounded by residues on β1, α4, the N-terminal end of β2 and θ2 and the β5-β6 loop (Fig. 6). His25 forms three hydrogen bonds to the pyrimidine (Fig. 9A). The imidazole ND1 and main-chain amide donate hydrogen bonds to the nucleotide O2 and N3 respectively, whereas the carbonyl accepts a hydrogen bond from N4. N4 further interacts with the main-chain carbonyl of Lys145. The cytosine position is also stabilized by hydrophobic contacts with the side chains of Tyr24 and Tyr175, the latter forming π–π stacking interactions with the cytidine. Tyr175 is replaced in *Ec*IspE by a phenylalanine, a conservative substitution and we note a degree of flexibility for this side chain because it adopts slightly different conformations in the three *Aa*IspE complex structures and with elevated thermal parameters compared with the more buried Tyr24. A large aromatic residue at this position in the active site may act as a gatekeeper during substrate binding and product release.

The ribose is solvent accessible and water-mediated contacts link O2′, Ser176 OG and the main-chain carbonyl of Gly172. The α-phosphate accepts a hydrogen bond donated from the main-chain amide of Ala129, and the β-phosphate interacts with Ser128 OG, the amide of Asp130 and also, via a solvent-mediated bridge, with Ser97 OG (not shown). The erythritol moiety of CDPME protrudes into a cavity in which catalytic residues Lys9 and Asp130 are placed. An extensive network of hydrogen-bonding interactions positions key residues and functional groups on substrate to support catalysis. Lys9 NZ is held in place by a hydrogen bond with Asn11 OD1. Three additional hydrogen bonds serve to position Asn11. OD1 also accepts a hydrogen bond donated from the main-chain amide of Gly237 and ND2 donates hydrogen bonds to Thr29 OG1 and to the substrate O1M hydroxyl. O1M forms a bidentate interaction with Asp130 OD1 and OD2. The O3M hydroxyl interacts with Tyr175 OH and a well-ordered water molecule that, in turn, forms hydrogen bonds with the main-chain carbonyl of Lys145 and Asp130 OD1 (data not shown). The methyl substituent on C2 is directed towards a hydrophobic pocket formed by the side chains of Tyr175 and Leu208.

The O2M group, where phosphate addition occurs, participates in hydrogen bonds with Lys9 NZ, Asp130 OD2 and a water molecule (not shown) that in turn associates with the main-chain amide and OG of Ser238, and β-phosphate of ADP. The placement and interactions formed by O2M are consistent with a mechanism that involves polarization of the hydroxyl group and Asp130 acting as a general base in proton abstraction to generate a nucleophile. This can then attack and acquire the reactive γ-phosphate of ATP with Lys9 NZ, and Ser238 amide and OG groups placed to stabilize the transition state. An overlay of the *Ec*IspE AMPPNP complex [13] with *Aa*IspE places the γ-phosphate group towards the catalytic residues Lys9 and Asp130 in a position consistent with the proposed mechanism (not shown).

In complex I, CDP displays a single conformation in molecule A and three in molecule B (not shown). Such conformational freedom, distinct from the ordered substrate, may reflect loss of the methylerythritol component and the numerous stabilizing interactions that it forms deep within the enzyme active site. The cytosine moieties of the four CDP molecules interact with Tyr24, His25, Lys145 and Tyr175, as described previously, with differences (not shown) confined to the orientation and binding modes of the solvent-exposed ribose and phosphate groups. In molecules A and B, the ribose O2′ interacts with the carbonyl of Gly23 via a water molecule. In addition, in molecule A, this group also interacts with the Gly172 carbonyl and Ser176 OG via water molecules; the α-phosphate interacts with Ser138 OG via a water molecule and the β-phosphate forms a direct hydrogen bond with Tyr24 OH. One CDP conformer in molecule B is similar to that observed in molecule A. In the second conformer in molecule B the α-phosphate occupies a similar position but the β-phosphate group is directed out of the active site to interact with both Tyr24 OH and the carbonyl of Ile127. This latter interaction suggests that protonation of the phosphate has occurred. In the third conformer the α-phosphate interacts with the backbone amide of Gly172, the β-phosphate group with the Ser169 carbonyl, Thr171 amide and OG1.

### The ATP-binding site

ADP binds in a cavity formed by the N-terminal domain adjacent to the CDPME-binding site (Figs 6 and 9B). The ADP is surrounded by residues in β2, at the N-terminal end of α1 and α2 and in the β4–α2 and β3–θ1 loops. ATP generally binds to kinases with the purine in an *anti* conformation with respect to the ribose. There are, however, two exceptions in which the energetically less favourable *syn* conformation is observed, *Ec*IspE [13] and the related homoserine kinase [21]. In *Aa*IspE the *syn* conformation is also observed with the adenine moiety placed in a hydrophobic cleft surrounded by aliphatic side chains of Ile53, Ile55, Leu61, Ile83 and Lys85 with contributions from Pro89 and Gly96. The purine conformation is stabilized by hydrogen-bonding contacts. Two asparagines (Asn60 and Asn99), themselves positioned by a network of hydrogen-bonding interactions, are placed to accept hydrogen bonds donated by adenine N6. Asn60 OD1 interacts with the amide of Val62, ND2 forms water-mediated contacts with Val49 and Ile55 carbonyl groups. Asn99 OD1 accepts hydrogen bonds from adenine N6 and Lys85 NZ, ND2 interacts with Ser6 OG and Asp38 OD2. Adenine N1 also interacts with Lys85 NZ, which is held in place by interaction with Asp38 and via a water molecule with the ADP α-phosphate. The amide of Leu61 donates a hydrogen bond to adenine N7. The ribose and phosphate groups of the ADP are solvent accessible and the α–β-linking oxygen forms a water-mediated contact with the Pro88 carbonyl and Leu93 amide. The β-phosphate accepts hydrogen bonds donated by amides of Gly92 and Gly94, and is linked to the Gly239 and Ser169 carbonyls, Lys9, Asp130 and Ser238 side-chain groups via water molecules. Another water-mediated interaction is formed between Thr171 OD1 and the ADP β-phosphate. In *Aa*IspE and *Ec*IspE complex structures, the adenine and ribose moieties bind in a similar position with highly conserved hydrogen-bonding interactions. The phosphate groups, however, adopt different conformations in the two structures, which may simply reflect the difference between AMP-PNP present in the *Ec*IspE structure and ADP in *Aa*IspE (not shown). The amide of Gly97 binds to the ADP α-phosphate in *Aa*IspE, whereas the corresponding Gly107 of *Ec*IspE coordinates to the β-phosphate. Furthermore, the Gly103 amide binds to the AMP-PNP γ-phosphate in *Ec*IspE, whereas it is the β-phosphate that binds to the amide of the corresponding Gly92 in *Aa*IspE.

Based on comparisons of the apo-structure of *Tt*IspE with the Mg^2+^-bound crystal structures of homoserine kinase [21] and mevalonate kinase [22] Wada *et al.* [14] suggested that a metal ion would bind to Ser95 and Asp125 (corresponding to Ser97 and Asp130 in *Aa*IspE). To investigate metal-ion binding in the catalytic site, *Aa*IspE was co-crystallized in the presence of 20 mm magnesium chloride or manganese chloride together with ADP or AMP-PNP and several crystal structures determined at medium resolution (data not shown). However, despite the clear dependence of activity on divalent cations, and in similar fashion to *Ec*IspE [13], we were unable to identify the position of any ordered metal ions in the active site of *Aa*IspE.

### Synthesis and binding of compound 1

**Scheme 1:**
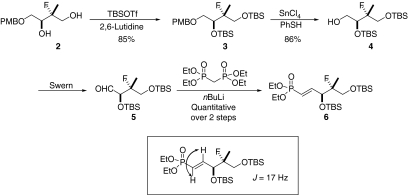
The outline for synthesis of the protected phosphonate ester compound 6.

The conjugated fluoro-MEP derivative compound **1** was designed as a fragment mimic of the β-phosphate and erythritol moiety of the substrate CDPME. The compound was expected to be a competitive inhibitor and to bind in a similar position to that observed for that component of the substrate represented in complex II. It was first necessary to determine a synthetic method for the molecule. Synthesis of **1** began with the enantiomerically pure fluorodiol **2**, which was protected to afford the triol **3** (Scheme 1). Removal of the *para*-methoxybenzyl (PMB) was achieved by use of Yu’s Lewis acid catalyzed conditions to give **4**. The free primary alcohol was then oxidized under Swern conditions and the resulting aldehyde **5** subjected to a Horner–Wadsworth Emmons olefination to afford the *E*-conjugated phosphonate ester **6** in quantitative yield and as a single double bond isomer. Double bond geometry was assigned by ^1^H NMR analysis (*J* =17 Hz). The silyl protecting groups were removed under standard trifluoroacetic acid (TFA) conditions to give the free 1,3-diol **7** in good yield.

**Scheme 2:**
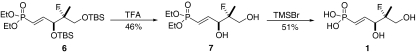
Deprotection of compound 6 to produce compound 1.

Having unmasked the diol functionality, it was then necessary to free the vinyl phosphate. Treatment of **7** with bromotrimethylsilane (TMSBr) generated the desired enantiomerically pure vinyl phosphate **1** in reasonable yield (Scheme 2).

**Fig. 10:**
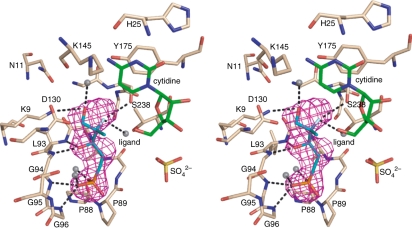
Stereo-view of compound 1 bound in the active site. The map, purple mesh, has been calculated with coefficients |*F*_o_−*F*_c_| and α_c_, and contoured at 2.5σ. *F*_o_ represents the observed structure factors, *F*_c_ represents the calculated structure factors and α_c_ represents the calculated phases for which ligand contributions were omitted. The model phases were generated from the final refined model. The ligand atoms are shown as sticks and coloured C, cyan; O, red; F white; P, orange. Nearby is a sulfate (S yellow, O red). Water molecules are shown as grey spheres. Potential hydrogen bonding interactions between 1 and the enzyme are shown as black dashed lines, and cytidine C atoms are green. Residues important in binding 1 within the active site are labelled.

We were unable to detect any ligand binding or enzyme inhibition of *Aa*IspE by **1** (data not shown), suggesting low affinity for the enzyme. However, we were able to determine the crystal structure of the complex where the ligand was bound to one molecule of the asymmetric unit following soaking of a crystal in a solution of **1**. Analysis reveals that **1** binds with the phosphonate occupying a similar position to that observed for the ADP/ATP α-phosphate not the β-phosphate of substrate (Fig. 10). The phosphate is positioned by hydrogen bonding to the main-chain amides Gly95 and Gly96, and water-mediated interactions with the carbonyl of Pro88, the amide of Leu93 together with the amide and OG groups of Ser97. The ligand C3 OH interacts with the amide groups of Leu93 and Gly94, the fluorine substituent at C4 with Ser238 OG and, via a water molecule, also with the carbonyl groups of Ser169 and Gly239, and Thr171 OD1. The C5 hydroxyl makes one hydrogen bond to a water molecule and is 3.2 Å from Lys9 NZ, although in the latter case the geometry is not optimal for a hydrogen bonding interaction.

### AaIspE represents a template for inhibitor development

*M. tuberculosis* is a particularly important human pathogen. Approximately one-third of the world’s population is infected with this organism, the causative agent of tuberculosis, which was responsible for an estimated 1.6 million deaths in 2005 [23]. The need for novel chemotherapeutics in the treatment of *M. tuberculosis* infection is clearly demonstrated by its high infectivity rate, the prolonged and extensive therapy requirements and the increase in drug-resistant forms. New targets for drug discovery research are urgently sought and the non-mevalonate pathway provides potentially valuable enzymes for such use [24,25]. Our studies of *Mtb*IspE have been hampered by poor solubility of the recombinant enzyme and a failure to obtain crystals (data not shown). To investigate whether the *Aa*IspE structure represents a model system to support inhibitor development we compared the sequences (Fig. 7).

In the above sections we detailed 17 *Aa*IspE residues that interact with CDPME (directly or via water molecules) or that contribute to the placement of functional groups that bind the substrate. Of these, 11 residues are strictly conserved. Five of the remainder are not significant because either main-chain groups interact by hydrogen-bonding interactions (Ala129 → Ser, Lys145 → Arg, Gly172 → Ser), there are solvent bridges involved (Ser97 → Ala, Ser176 → Asn) or in the case of the Ser/Asn change a direct interaction might occur. The final difference is Ser128 → Gly. Ser128 OG interacts with the β-phosphate of CDPME. Truncation of the side chain to glycine might allow a water molecule to compensate for the loss of such an interaction. Similar observations apply when comparing the ATP-binding site. Of the 21 residues discussed above, 10 are strictly conserved and they are mainly those residues that make direct interactions with ATP. Three changes (Ala91 → Gly, Leu93 → Met, Ser169 → Leu) involve residues that interact using main-chain groups, or that use side chains to form the hydrophobic purine binding pocket, e.g. Pro89 → Val. Two alterations are of note. In *Aa*IspE, the side-chain carbonyl groups of Asn60 and Asn99 accept hydrogen bonds from adenine N7 (Fig. 10B). In the sequence alignment shown in Fig. 8, Asn60 and Asn99 correspond to Arg69 (a major alteration) and Asp109 (a conservative change) of *Mtb*IspE, respectively. There may be some difference in *Mtb*IspE such that Asn70 is actually placed to provide the same function as Asn60 of *Aa*IspE.

## Conclusions

We purified soluble recombinant *Aa*IspE in high yield, optimized the enzyme assay and kinetically characterized the enzyme, identified conditions that provide a reliable source of well-ordered crystals and determined a series of structures with physiologically relevant ligands. We designed and synthesized a fragment to mimic a component of the substrate and provide a template for inhibitor development. Surprisingly, when soaked into a crystal the ligand adopts a position that overlays where cofactor binds rather than substrate. The collection of structures serves to inform on aspects of specificity and reactivity of IspE. Sequence–structure comparisons indicate that the active site and interactions with ligands are highly conserved and that *Aa*IspE represents a suitable model for orthologues derived from human pathogens including the important *M. tuberculosis*. We have shown that the crystals are suitable for soaking in small ligands and as such can support a fragment and structure-based approach to underpin drug discovery targeting important human diseases [26,27].

## Experimental procedures

### Cloning, expression and purification

The gene-encoding sequence of *Aa*IspE was identified in the UniProt Database (entry O67060) and cloned from genomic DNA by PCR using the following primers (*Nde*I and *Xho*I restriction sites are underlined): forward: CATATGATAAAGGTTCTATCACCCGCG, reverse: CTCGAGTTAGAGTTCTAGCTCTACAAC. The gene was inserted into the pCR blunt II TOPO vector (Invitrogen, Carlsbad, CA), and then subcloned into the pET15b vector (Novagen, Darmstadt, Germany) which was heat-shock transformed into *E. coli* BL21 (DE3). Cells were cultured in Luria–Bertani broth containing ampicillin (100 mg·L^−1^) at 37 °C. Protein production was induced overnight at 30 °C by addition of 1 mm isopropyl-β-d-thiogalactoside. Following centrifugation, cells were resuspended in 50 mm.

Tris/HCl pH 8.5, 250 mm NaCl and 3 mmβ-mercaptoethanol and lysed using a French Press. Cell debris was removed by centrifugation at 40 000 ***g*** and the supernatant containing soluble protein was loaded onto a 5 mL Ni^2+^ HisTrap column (GE Healthcare, Amersham, UK). *Aa*IspE was eluted with a linear imidazole gradient. The His_6_ tag was removed by digestion with thrombin for 6 h at 4 °C and the sample dialysed against 50 mm Tris/HCl pH 8.5, 50 mm NaCl and 1 mm dithiothreitol. This was then applied to a 5 mL HiTrapQ HP anion-exchange column (GE Healthcare) and *Aa*IspE eluted with a linear NaCl gradient. After dialysis against 10 mm Tris/HCl pH 8.5, 20 mm NaCl and 1 mm dithiothreitol, the protein was concentrated with centrifugal force (VivaSpin20, molecular mass cut-off 10 kDa; Vivascience, Littleton, MA). Concentration was estimated using a theoretical extinction coefficient of 0.898 g·L^−1^ at 280 nm. The purity of the sample was assessed by SDS/PAGE and MALDI-TOF MS.

To produce the SeMet derivative, the *Aa*IspE/pET15b plasmid was transformed into the *E. coli* methionine auxotroph B834Met^−^ (Stratagene, La Jolla, CA). Cultures were grown in M9 minimal media supplemented with 4 mg·L^−1^ FeSO_4_.7H_2_O and 0.5 g·L^−1^ each of adenine, guanosine, thymine and uracil. We added 40 mg·L^−1^ of all amino acids with the exception of methionine, which was replaced by 100 mg·L^−1^ SeMet (Sigma Aldrich, Poole, UK). Protein production and purification were carried out as described for the native protein. Full incorporation of SeMet was confirmed by MALDI-TOF MS.

### Photometric assay for IspE enzyme activity

Assay mixtures contained 100 mm Tris/HCl, pH 8.5, 20 mm KCl, 10 mm MgCl_2_, 0.45 mm NADH, 2 mm ATP, 4 mm phosphoenolpyruvate, 1 mm CDPME, 8 U pyruvate kinase, 9 U lactate dehydrogenase and 3 μg *Aa*IspE. The assay mixtures were incubated at 37 °C, and the reaction monitored photometrically at 340 nm.

### NMR assay for IspE enzyme activity

Assay mixtures contained 100 mm Tris/HCl, pH 8.5, 2 mm MgCl_2_, 2.5 mm [1,3,4-^13^C_3_]CDPME, 8 mm ATP, 23–100 μg *Aa*IspE, 5 mm divalent metal ions and 10% (v/v) D_2_O in a total volume of 500 μL. The mixtures were incubated at 37 °C for 30–60 min, terminated by the addition of EDTA to a final concentration of 25 mm and analysed by ^13^C NMR spectroscopy. This assay was used to determine the temperature and metal-ion dependence.

### Synthesis of conjugated the fluoro-MEP analogue

All reactions were performed under an inert argon atmosphere. Anhydrous tetrahydrofuran, dichloromethane (DCM) and diethyl ether were obtained through a Pure Solv 400-5MD solvent purification system (Innovative Technology, Inc., Cleveland, OH). All reagents purchased from commercial sources were used without further purification, unless otherwise stated. Solvents were evaporated under reduced pressure at 35 °C using a Büchi Rotavapor. Proton magnetic resonance spectra (^1^H NMR) were recorded at 400 MHz using Bruker Avance400 or Bruker DPX400 instruments. Chemical shifts, δ are reported in parts per million (p.p.m.), and are referenced to the residual solvent peak. The order of citation in parentheses is (a) number of equivalent nuclei (by integration), (b) multiplicity (s, singlet; d, doublet; t, triplet; q, quartet; m, multiplet), (c) coupling constant, with *J* quoted in Hertz to the nearest 0.5 Hz and (d) assignment. Carbon magnetic resonance spectra (^13^C NMR) were recorded at 101 MHz using Bruker Avance400 or Bruker DPX400 instruments. Chemical shifts are quoted in p.p.m. and are referenced to the residual solvent peak. The assignment is quoted in parentheses. Fluorine magnetic resonance spectra (^19^F NMR) were recorded at 376 MHz using a Bruker Avance400 instrument referenced to CFCl_3_. Phosphorus magnetic resonance spectra (^31^P NMR) were recorded at 162 MHz using a Bruker Avance400 instrument and are referenced to external phosphoric acid (δ 0.00 p.p.m.). High-resolution mass spectra were recorded on a JEOL JMS-700 spectrometer by electrospray ionization mass spectrometer operating at a resolution of 15 000 full widths at half height.

Infrared spectra were recorded as thin films on NaCl plates using a JASCO FT/IR 4100 instrument. Only significant absorptions (υ_max_) are reported in wavenumbers (cm^−1^) with the following abbreviations to describe absorption intensity: w, weak; m, medium; s, strong; and br, broad. Optical rotations, [α]_D_ values were recorded on a Rudolph Research analytical Autopol V automatic polarimeter at 25 °C, with the concentration and solvent reported in brackets. Flash chromatography was carried out using silica gel (Apollo Scientific Gel 60, 40–63 μm) as the stationary phase. TLC was carried out using aluminium sheets pre-coated with silica (Merck Silica Gel 60 F_254_). Detection was under a UV light source (λ_max_ 254 nm) or through staining with anisaldehyde or potassium permanganate solution, with subsequent heating. *R*_F_ (retention factor) values are quoted to ± 0.05. The specific details and characterization of compounds are given below and in Ghilagaber *et al.* [28].

#### 1-[2,4-Bis-(*tert*-butyl-dimethyl-silanyloxy)-3-fluoro-3-methyl-butoxymethyl]-4-methoxy-benzene, 3

A −78 °C solution of 2-fluoro-4-(4-methoxy-benzyloxy)-2-methyl-butane-1,3-diol **2** (0.27 g, 1.0 mm) in anhydrous DCM (20 mL) was treated with trimethylsilyl trifluoromethanesulfonate (0.7 mL, 4.0 mm) and 2,6-lutidine (0.5 mL, 4.20 mm). The reaction mixture was stirred for 1.5 h at −78 °C, allowed to warm to room temperature, the aqueous layer was extracted with DCM (3 × 20 mL), the combined organic layers were dried over anhydrous magnesium sulfate and the solvent was removed under reduced pressure. The crude product was purified on a silica gel column using ethyl acetate/petroleum ether (15 : 85) to yield 510 mg 1-[2,4-bis-(*tert*-butyl-dimethyl-silanyloxy)-3-fluoro-3-methyl-butoxymethyl]-4-methoxy-benzene **4** (quantitative) as a clear oil.

^1^H NMR (400 MHz, CDCl_3_): δ = 7.26 (2H, d, *J* = 8.6 Hz, Ar**H**), 6.88 (2H, d, *J* = 8.7 Hz, Ar**H**), 4.43 (2H, s, PhC**H**_**2**_), 4.08 (1H, td, *J* = 7.6, 2.2 Hz, C**H**), 3.81 (3H, s, OC**H**_**3**_), 3.70 (1H, d, *J* = 6.2 Hz, CH_A_**H**_**B**_), 3.65 (2H, m, C**H**_**2**_), 3.42 (1H, ddd, *J* = 9.6, 7.1, 2.0 Hz, C**H**_**A**_H_B_), 1.22 (3H, d, *J*_HF_ = 22.0 Hz, CFC**H**_**3**_), 0.90 (9H, s, 3 × C**H**_**3**_), 0.88 (9H, s, 3 × C**H**_**3**_), 0.08 (6H, s, 2 × C**H**_**3**_), 0.06 (3H, s, C**H**_**3**_), 0.05 (3H, s, C**H**_**3**_).

^13^C NMR (100 MHz, CDCl_3_): δ = 159.2 (Ar**C**), 130.6 (Ar**C**), 129.4 (2 × Ar**C**H), 113.8 (2 × Ar**C**H), 98.3 (d, *J*_CF_ = 172 Hz, **C**FCH3), 73.0, 72.8, 72.1, 65.9 (d, *J*_CF_ = 24.4 Hz), 55.4 (O**C**H_3_), 26.0 (6 × **C**H_3_), 18.4, 18.1, 17.5 (d, *J*_CF_ = 22.1 Hz), −4.1 (**C**H_3_), −5.1 (**C**H_3_), −5.2 (**C**H_3_), −5.3 (**C**H_3_).

^19^F NMR (400 MHz, CDCl_3_): δ = −159.20

*R*_F_ [diethyl ether/petroleum ether (1 : 1)] 0.51

HRMS (ESI) calcd for C_25_H_47_FO_4_Si_2_ (M + Na^+^): 486.2997. Found *m/z* 509.3300.

[α]_D_ = −5.3 (c 1.15; DCM).

IR: υ_max_ (film) 2857 cm^−1^ (s, O-CH_3_) and 1253 cm^−1^ (s, C-F).

#### 2,4-Bis-(*tert*-butyl-dimethyl-silanyloxy)-3-fluoro-3-methyl-butan-1-ol, 4

A solution of 1-[2,4-bis-(*tert*-butyl-dimethyl-silanyloxy)-3-fluoro-3-methyl-butoxymethyl]-4-methoxy-benzene **3** (0.14 g, 0.29 mm) in anhydrous DCM (2.5 mL) was cooled to −78 °C. Thiophenol (0.04 mL, 0.36 mm) was added, immediately followed by tin (IV) chloride (0.32 mL, 1.0 m in DCM). The resulting mixture was then stirred for exactly 20 min and the reaction was quenched with saturated sodium hydrogen carbonate (10 mL). The aqueous layer was then extracted with DCM (3 × 20 mL), the combined organic layers were dried over anhydrous magnesium sulfate and the solvent was removed under reduced pressure. The crude residue was purified on a silica gel column using ethyl acetate/petroleum ether (1%) to yield 90 mg 2,4-bis-(*tert*-butyl-dimethyl-silanyloxy)-3-fluoro-3-methyl-butan-1-ol **4** (86%) as a clear oil.

^1^H NMR (400 MHz, CDCl_3_): δ = 3.96 (1H, m, C**H**), 3.70 (4H, m, 2 × C**H**_**2**_), 2.06 (1H, td, *J* = 6.3, 1.7 Hz, O**H**), 1.28 (3H, d, *J*_HF_ = 22.4 Hz, CFC**H**_**3**_), 0.91 (9H, s, 3 × C**H**_3_), 0.90 (9H, s, 3 × C**H**_**3**_), 0.07 (6H, s, 2 × C**H**_**3**_), 0.11 (3H, s, C**H**_**3**_), 0.12 (3H, s, C**H**_**3**_).

^13^C NMR (100 MHz, CDCl_3_): δ = 95.9 (**C**FCH_3,_*J*_CF_ =172 Hz), 74.0 (d, *J*_CF_ = 26.2 Hz), 65.5 (d, *J*_CF_ = 27.1 Hz), 63.3 (d, *J*_CF_ = 5.7 Hz), 26.0 (6 × **C**H_3_), 18.5, 18.3, 17.9, −5.3 (**C**H_3_), −5.2 (**C**H_3_), −4.8 (**C**H_3_), −4.4 (**C**H_3_).

^19^F NMR (376 MHz, CDCl_3_): δ = −157.8

*R*_F_ [ethyl acetate/petroleum ether (2%)] 0.14

[α]_D_ = −2.6 (c 1.16, CH_2_Cl_2_)

IR: υ_max_ (film) 1255 cm^−1^ (s, C-F)

#### (*E*)-[3,5-Bis-(*tert*-butyl-dimethyl-silanyloxy)-4-fluoro-4-methyl-pent-1-enyl]-phosphonic acid diethyl ester, 6

Dimethyl sulfoxide (90 μL, 1.30 mm) was added to a −78 °C solution of oxalyl chloride (60 μL, 0.65 mm) in anhydrous DCM (5 mL) and the resulting solution was stirred for 15 min. A solution of 2,4-bis-(*tert*-butyl-dimethyl-silanyloxy)-3-fluoro-3-methyl-butan-1-ol **4** (0.12 g, 0.33 mm) in anhydrous DCM (5 mL) was added drop wise and the reaction mixture was stirred for a further 30 min at −78 °C. Triethylamine (0.30 mL, 2.15 mL) was then added and the reaction was allowed to warm to room temperature and left to stir for 1 h. A 2 : 1 solution of diethylether and saturated aqueous sodium hydrogen carbonate (45 mL) was added and the aqueous layer was extracted with diethylether (3 × 20 mL). The combined organic layers were dried over anhydrous magnesium sulfate and the solvent removed under reduced pressure. The crude residue (0.15 g) of 2,4-bis-(*tert*-butyl-dimethyl-silanyloxy)-3,3-dimethyl-butyraldehyde **5** was used straight away without further purification. ^1^H NMR (400 MHz, CDCl_3_): δ = 9.56 (1H, dd, *J* = 5.1, 1.3 Hz, C(O)**H**), 4.15 (1H, dd, *J* = 10.8, 1.3 Hz, C**H**), 3.82 (1H, t, *J =*10.0 Hz, C**H**_**A**_H_B_), 3.49 (1H, dd, *J* = 13.9, 10.0 Hz, CH_A_**H**_**B**_), 1.32 (3H, d, *J* = 22.9 Hz, CFC**H**_**3**_), 0.90 (9H, s, 3 × C**H**_3_), 0.87 (9H, s, 3 × C**H**_3_), 0.05 (12H, m, 4 × C**H**_3_). *R*_F_ [petroleum ether] 0.26

Tetraethyl methylenediphosphonate (0.16 mL, 0.64 mm) in dry tetrahydrofuran (6 mL) was cooled to −78 °C and stirred for 5 min. *n*BuLi (0.19 mL, 2.5 m sol. in hexane) was added to the phosphate solution and the mixture stirred for 30 min at −78 °C. A solution of the crude butyraldehyde **5** (0.15 g, 0.41 mm) in anhydrous tetrahydrofuran (7 mL) was added, and the resulting solution was stirred for 1 h at −78 °C and then a further 1.5 h at room temperature. The reaction was quenched with 10% ammonium chloride solution (20 mL) and the aqueous layer extracted with ethyl acetate (3 × 20 mL). The combined organic layers were dried over anhydrous magnesium sulfate and the solvent removed under reduced pressure. The product was purified on a silica gel column using ethyl acetate/petroleum ether (2%) to yield 160 mg of the desired [3,5-bis-(*tert*-butyl-dimethyl-silanyloxy)-4-fluoro-4-methyl-pent-1-enyl]-phosphonic acid diethyl ester **6** (quantitative yield over both steps) as a clear oil.

^1^H NMR (400 MHz, CDCl_3_): δ = 6.80 (1H, dddd, *J* = 17.2, 4.3, 2.0 Hz, *J*_HP_ = 22.1 Hz, C**H**), 5.93 (1H, ddd, *J* = 17.2, 1.5, *J*_HP_ = 22.1 Hz, C**H**), 4.54 (1H, m, C**H**), 4.06 (5H, m, 2 × C**H**_**2**_ and C**H**_**A**_H_B_), 3.63 (1H, m, CH_A_**H**_**B**_), 1.28 (6H, t, *J* = 7.1 Hz, CFC**H**_**3**_), 1.13 (3H, d, *J*_HF_ = 21.8 Hz, CFC**H**_**3**_), 0.87 (9H, s, 3 × C**H**_3_), 0.86 (9H, s, 3 × C**H**_**3**_), 0.05 (3H, s, C**H**_3_), 0.03 (3H, s, C**H**_3_), 0.02 (3H, s, C**H**_3_), −0.01 (3H, s, C**H**_3_).

^13^C NMR (100 MHz, CDCl_3_): δ = 151.0 (d, *J*_CP_ = 5 Hz), 119.8 (d, *J*_CP_ = 186.5 Hz), 97.5 (d, *J*_CF_ = 176.9 Hz), 72.5 (d, *J*_CF_ = 21.2 Hz), 72.2 (d, *J*_CF_ = 21.3 Hz), 65.6, 61.8 (appt. t, *J*_CP_ = 4.9 Hz), 26.0 (3 × **C**H_3_), 25.9 (3 × **C**H_3_), 18.4, 18.1, 16.8 (d, *J*_CF_ = 22.2 Hz), 16.5, 16.4, −4.3 (**C**H_3_), −5.1 (**C**H_3_), −5.2 (**C**H_3_), −5.4 (**C**H_3_).

^19^F NMR (376MHz, CDCl_3_): δ = −160.35

^31^P NMR (162MHz, CDCl_3_): δ = 18.48

*R*_F_ [ethyl acetate/petroleum ether (3 : 7)] 0.22

HRMS (CI^+^) calcd for C_22_H_49_FO_5_PSi_2_ (M + H)^+^: 499.2840. Found *m/z* 499.2839.

[α]_D_ = −10.6 (c 1.12, CHCl_3_).

IR: υ_max_ (film) 1258 cm^−1^ (s, P=O).

#### 4-Fluoro-3,5-dihydroxy-4-methyl-pent-1-enyl)-phosphonic acid diethyl ester, 7

A solution of [3,5-bis-(*tert*-butyl-dimethyl-silanyloxy)-4-fluoro-4-methyl-pent-1-enyl]-phosphonic acid diethyl ester **6** (0.44 g, 0.88 mm) in anhydrous DCM (2 mL) was treated with 90% trifluoroacetic acid (4.08 mL, 54.93 mm). The resulting solution was stirred at room temperature for 30 min and the volatile solvents were then removed on a rotary evaporator. The product was purified on a silica gel column using DCM/methanol (9 : 1 v/v) to yield 110 mg of the desired 4-fluoro-3,5-dihydroxy-4-methyl-pent-1-enyl)-phosphonic acid diethyl ester **7** (46%) as a clear oil.

^1^H NMR (400 MHz, CDCl_3_): δ = 6.87 (1H, ddm, *J* = 20.8, 17.2 Hz, CH), 6.14 (1H, ddm, *J* = 21.0, 17.3 Hz, CH), 4.59 (2H, m, CH_2_ and 2 × OH), 4.07 (4H, m, 2 × CH_2_), 3.74 (2H, m, CH_2_), 1.32 (6H, td, *J* = 7.0 Hz, *J*_HP_ = 0.9 Hz, 2 × CH_3_), 1.17 (3H, d, *J*_HF_ = 22.2 Hz, CFCH_3_).

^13^C NMR (100 MHz, CDCl_3_): δ = 150.8 (d, *J*_CP_ = 3.9 Hz) 118.5 (d, *J*_CP_ = 188 Hz), 97.0 (d, *J*_CF_ = 173.8 Hz), 71.9 (d, *J*_CF_ = 71.9 Hz), 71.6 (d, *J*_CF_ = 71.6 Hz), 66.1 (d, *J*_CF_ = 23.0 Hz, CH_2_), 62.4 (appt. t, *J* = 5.9 Hz, -CH_2_CH_3_), 17.1 (d, *J*_CF_ = 22.4 Hz, -CFCH_3_), 16.4 (d, *J*_CP_*=*6.4 Hz, 2**×**−CH_2_CH_3_).

^31^P NMR (162 MHz, CDCl_3_): δ = 19.2

^19^F NMR (376 MHz, CDCl_3_): δ = −160

*R*_F_ [DCM/MeOH (9 : 1)] 0.37

HRMS (CI^+^) calcd for C_10_H_21_O_5_FP (M + H)^+^: 271.1111. Found *m/z* 271.1107.

[α]_D_ = −40.1 (c 0.94, CHCl_3_)

IR: υ_max_ (film) 3403 cm^−1^ (br, O-H)

#### (4-Fluoro-3,5-dihydroxy-4-methyl-pent-1-enyl)-phosphonic acid, 1

A solution of 4-fluoro-3,5-dihydroxy-4-methyl-pent-1-enyl)-phosphonic acid diethyl ester **7** (0.14 g, 0.52 mm) in anhydrous DCM (2 mL) was treated with TMSBr (0.46 mL, 3.49 mm) and stirred for 20 h at room temperature. The volatile solvents were removed under vacuum and the resultant residue was treated with a 1 : 1 solution of ethanol/water (4 mL) for 30 min and the solvents were once again removed under reduced pressure. This process was repeated three times and the crude residue was purified on a silica gel column using methanol/DCM (1 → 10%) to yield 60 mg of the desired (4-fluoro-3,5-dihydroxy-4-methyl-pent-1-enyl)-phosphonic acid **1** (51%) as a white solid.

^1^H NMR (400 MHz, MeOD): δ = 6.80 (1H, ddm, *J* = 20.9, 17.3 Hz, CH), 6.19 (1H, dd, *J* = 20.4, 17.8 Hz, CH), 4.95 (4H, s, 4**×**OH), 4.48 (1H, m, CH), 3.71 (2H, m, CH_2_), 1.22 (3H, d, *J*_HF_*=*22.1, CFCH_3_).

^13^C NMR (100 MHz, MeOD): δ = 148.6, 122.1 (d, *J*_CP_ =185.4 Hz), 99.59 (d, *J*_=_174.2 Hz), 72.4 (d, *J*_CF_ = 72.5 Hz), 72.2 (d, *J*_CF_ = 72.3 Hz), 66.2 (d, *J*_CF_ = 22.7 Hz, CH_2_), 16.9 (d, *J*_CF_ = 22.8 Hz, CH_3_).

^31^P NMR (162 MHz, MeOD): δ = 16.2

^19^F NMR (376 MHz, MeOD): δ = **−**164.3

*R*_F_ [DCM/MeOH (9 : 1)] 0.05

HRMS (FAB^+^) calcd for C_6_H_13_O_5_FP (M + H)^+^: 215.0485. Found *m/z* 215.0488.

[α]_D_ = **−**21.4° (c 0.46, CH_3_OH)

IR: υ_max_ (film) 3410cm^−1^ (br, O–H)

### Crystallization

*Aa*IspE was crystallized by vapor diffusion in hanging drops assembled from 2 μL reservoir and 2 μL protein solution at a concentration of 10 mg·mL^−1^. A reservoir of 0.1 m Mes pH 6.5, 0.20 m NaCl and 1.50 m (NH_4_)_2_SO_4_ was used for the native protein. Crystals of SeMet *Aa*IspE grew with a reservoir of 0.1 m Mes pH 6.5, 0.50 m NaBr and 1.55 m (NH_4_)_2_SO_4_. Triangular prisms (maximum dimension 0.4 mm) grew after a few days at 18 °C. SeMet and native *Aa*IspE (complex I) were co-crystallized with 4 mm AMP-PNP and 4 mm CDP. Crystals were also grown in the presence of 20 mm ADP, 20 mm CDPME2P and 10 mm MgCl_2_ (complex II). For soaking experiments to obtain complex III, a stabilizing solution of 0.1 m Mes pH 6.5, 0.2 m NaCl and 1.8 m (NH_4_)_2_SO_4_ was used and crystals soaked overnight with compound **1** at a concentration of 25 mm.

### Data collection and processing

Following cryo-protection for 30 s (1 : 1 mixture of reservoir with 100% 2-methyl-2,4-pentanediol) the crystals were cooled in a stream of nitrogen gas at −170 °C for data collection. Anomalous dispersion measurements were carried out on SeMet crystals using beam-line ID14-4 at the European Synchrotron Radiation Facility (Grenoble, France). A fluorescence scan around the Se K-edge identified the wavelengths for multiple-wavelength anomalous dispersion data collection. Data were collected at a peak: λ_1_ = 0.97947 Å, an inflection point: λ_2_ = 0.97958 Å and a high-energy remote point: λ_3_ = 0.97625 Å using a Q315 ADSC CCD detector. The SeMet crystals are cubic, in space group *P*2_1_3 with a unit cell edge of 137.8 Å and diffracted to 2.7 Å resolution.

Data were collected on beam-line ID23-1 at the E.S.R.F. on a Q315 ADSC CCD detector or in-house using a Rigaku 007 Micromax rotating-anode generator (Cu K_α,_λ = 1.5418 Å) operating at 30 mA and 40 kV, coupled to an R-AXIS IV^++^ dual image plate system. Data were processed and scaled with mosflm/scala [29,30] or denzo/scalepack [31]*.* Statistics are shown in Tables 3 and 4.

### Structure determination

The program solve [32] identified three of four selenium positions using data to 3.0 Å resolution and gave a figure-of-merit of 0.34 and a *Z*-score of 13. After density modification with resolve [33], the figure-of-merit increased to 0.72 and a correlation coefficient of 0.38 was obtained. resolve built an initial model of 446 (of 658) residues. The missing residues were included by interpretation of electron density using coot [34]. The model was used to initiate refinement of the ligand complexes using first molrep [35] then refmac [36]. Calculation of *R*_free_ used 5% of the data. Electron density and difference density maps, all σ_A_-weighted [37,38] were inspected and the model improved using coot. A subset of the data (5%) was set aside for calculation of *R*_free_. Electron and difference density map inspection, model manipulation together with addition of ligands including water molecules was carried out using coot interspersed with TLS (translation, libration, screw analysis) and maximum-likelihood restrained refinement with refmac. Strict non-crystallographic symmetry restraints were applied at first then released towards the end of each refinement. The quality of the model was assessed with procheck [39]*.* Figures were prepared with chemdraw (Adept Scientific, Letchworth Garden City, UK) pymol (http://pymol.sourceforge.net) [40] and aline (C. S. Bond, personal communication).

### Quaternary structure

Analytical gel-filtration chromatography used a Superose 10/300 analytical gel-filtration column (GE Healthcare) pre-equilibrated with buffer and calibrated with molecular mass standards, blue dextran (> 2000 kDa), BSA (67 kDa), carbonic anhydrase (29.5 kDa) and cytochrome *c* (12.5 kDa; GE Healthcare; data not shown). *Aa*IspE eluted at a volume of 94 mL corresponding to an approximate molecular mass of 30.0 kDa. Samples for analytical ultracentrifugation were prepared in the crystallization buffer (see above) at concentrations of 0.25, 0.5 and 1.0 mg·mL^−1^. Sedimentation velocity experiments were performed using a Beckman Coulter (Fullerton, CA, USA) XL-1 analytical ultracentrifuge (wavelength 280 nm, rotor AN50-TI at 45 000 r.p.m. and 20 °C). Samples were centrifuged simultaneously and *A*_280_ measurements taken at 5 min intervals for 16 h. The resultant data were analysed using sedfit and sednterp [41,42]. Analytical ultracentrifugation returned a sedimentation coefficient of 2.61s, which corresponds to a mass of ∼ 29.8 kDa (data not shown).

## Abbreviations:

*Aa*IspE: *Aquifex aeolicus* IspE
AMP-PNP: adenosine 5′-(β,γ-imino)triphosphate
CDPME: 4-diphosphocytidyl-2*C*-methyl-d-erythritol
CDPME2P: 4-diphosphocytidyl-2*C*-methyl-d-erythritol 2-phosphate
DCM: dichloromethane
*Ec*IspE: *Escherichia coli,* IspE
GHMP kinase: galacto, homoserine, mevalonate, and phosphomevalonate kinases
IspE: 4-diphosphocytidyl-2*C*-methyl-d-erythritol kinase
*Mtb*IspE: *Mycobacterium tuberculosis* IspE
SeMet: selenomethionine
*Tt*IspE: *Thermus thermophilus* IspE
